# Treating Schizophrenia with DOTS in Developing Countries: One Size Does Not Fit All

**DOI:** 10.1371/journal.pmed.0040281

**Published:** 2007-09-25

**Authors:** Renato Souza, Silvia Yasuda, Susanna Cristofani

Although DOTS is advocated as the best approach for global tuberculosis control, the variable success of this strategy [1] should help us in learning which problems we might face while adopting the same strategy for the treatment of schizophrenia in developing countries, as Dr. Farooq suggests [2].

Our experience of integrating mental health into primary health care in developing countries has taught us that some points stated in the five pillars of DOTS for tuberculosis cannot be totally transferred to the treatment of schizophrenia, unless some of their principles and weak points are addressed in advance [3]:

1. In developing countries, we face the challenge of integrating mental health knowledge into the skills of poorly qualified and over-burdened primary health care staff. Therefore, unless strong training and supervision capacity for staff at primary health care levels is developed, this obstacle won't be overcome.
2. Passive case finding, for a disease that provokes such high levels of disability, stigma, and human rights abuse as schizophrenia, is not appropriate in our view.
3. A standard treatment regimen needs to be overseen with caution if implemented for the treatment of schizophrenia, due to the need to adjust the dose of the antipsychotic based on patient response and side effects.
4. A regular supply of essential psychotropic medication is obligatory but non-existent in most developing countries, and when available does not extend to the primary health care level.
5. Monitoring and tracking patients under treatment is an enormous burden to overstretched primary health care systems, unless the community is heavily involved.

In Darfur-Sudan, due to the high level of mental health morbidity, Médecins Sans Frontières has been implementing a syndromic approach to the diagnosis of mental illnesses [4]

For the identification of patients with severe mental illness including schizophrenia, community health workers are trained to identify patients at the community level, using a locally developed case definition of severe mental illness based on existing local idiom for those conditions.

During a period of two months, we have identified 49 patients that were brought to the health clinic, where a medical assistant made the diagnosis and started the treatment. Community health workers provide therapeutic education to patients and caretakers and support them to continue the treatment within the community. All professionals are under the supervision of a mental health trainer.

Some patients were in such dramatic situations as being chained to their beds. Some had received several forms of traditional treatments without any success.

We firmly believe that unless a system is built where the community is involved, medical personnel receive training and supervision, and Ministries of Health commit to delivering a constant supply of drugs at the primary health care level, the attempt to use one or another strategy won't bring much relief to patients and families affected by this disease.

It was in 1974 that the World Health Organization recommended that mental health care be integrated at the primary health care level. The management of psychosis was identified as one of the priorities [5]. It is very unfortunate that in most of the places where Médecins Sans Frontières works, the majority of health workers usually neglect the needs of people with severe mental illness.
